# Precarity prevented or reinforced? Migrants' right to change employers in the recast of the EU Single Permit Directive

**DOI:** 10.3389/fsoc.2023.1267235

**Published:** 2024-01-11

**Authors:** Tesseltje De Lange, Mariella Falkenhain

**Affiliations:** ^1^Faculty of Law, Centre for Migration Law, Radboud University, Nijmegen, Netherlands; ^2^Research Unit Joblessness and Social Inclusion, Institute for Employment Research (IAB), Nuremberg, Germany

**Keywords:** labor migration, labor market policies, European Union, Single Permit Directive, third-country nationals, right to change employer, precarity

## Abstract

Labor migration policies within the European Union and its Member States typically address two conflictive labor market policy goals. They aim to attract and retain foreign workers in a situation of labor shortage, while at the same time protecting the national workforce from additional labor market competition. The balancing of these two goals is commonly resolved in favor of nationals and with fewer rights for migrant workers. It is precisely this nexus between migrant rights and the protection of the national workforce that is central to the understudied question of whether and under what conditions migrant workers from third countries (i.e. non-EU countries) may change their employer to quit low-quality work or exploitative employment, or for career reasons. Building on scholarly discussions on employer dependency and bureaucratic complexity as general sources of migrant precarity, as well as on international and EU law on the rights of migrant workers, this article presents three policy variations of the right to change employers currently in place in the European context. Thereby we fill a gap in the literature on labor migration, labor market regulation, and migrant workers' rights. To illustrate the mixed ambitions of EU institutions to reduce migrant precarity the article then presents and critically discusses the high-level negotiations over the recast of the EU Single Permit Directive 2011/98 that were centered around the right to change employers.

## 1 Introduction

In their labor migration policies the European Union (EU) and its Member States typically address two seemingly contradictory labor market policy goals: They aim to attract and retain non-EU migrant workers—also called third-country nationals (TCNs)—in a situation of labor shortage, while at the same time trying to protect the national workforce from additional labor market competition and to prevent (feared) wage undercutting. The balancing of these two goals is commonly resolved in favor of nationals by restricting labor market access and mobility for migrant workers. This is why especially migrant workers without formal qualifications often times end up in precarious employment and living situations and with uncertain legal staying perspectives (Ruhs and Martin, 2008; Lenard and Straehle, 2010; Fudge, 2012).

At three moments in the migration process, the nexus of migrant rights and the protection of the national workforce becomes particularly relevant: (1) during primary admission, (2) when admitted migrant workers (want to) change their employer (called visa portability in the US context) (Griffith, 2009), and (3) in case of unemployment and neediness. EU policies regulating the conditions of primary admission have been extensively studied by legal scholars (see for instance Farcy, 2020; Bregiannis, 2021; De Lange and Groenendijk, 2021; Minderhoud, 2021; De Lange and Vankova, 2022). The policies and logics of welfare support and its take-up by non-EU migrants have been examined in legal scholarship (e.g., Verschueren, 2016; Friðriksdóttir, 2017), but also in the fields of economics, social policy and sociology (for an overview, see Albertini and Semprebon, 2018). In the international literature on temporary labor migration, migrants' dependency on employers—a typical implication of a limited right to change jobs – has been widely acknowledged as an important source of precarity and as a legal disadvantage compared to natives who can freely choose their jobs (e.g., Fudge, 2012; Zou, 2015; Martin, 2021).

Migrant workers' right to change employer is a topic that commonly lacks political attention. Where migrants fear to lose not only their job but also their residence status, and given low levels of unionization among migrant workers, they rarely complain about exploitative employment relations or other difficulties stemming from employer dependency and structural insecurity (Berntsen, 2016; Campbell et al., 2019). This is how the trap that unfreedom to exit employment is setting in motion often remains under the radar. Recently, however, there was some international attention to the topic. At the time of writing, a Canadian workers' rights group filed an application for a class-action lawsuit in Quebec Superior Court to ban what they call “closed work permits”, permits which bind migrant workers to a specific employer. With the support of the UN special rapporteur on contemporary forms of slavery, they argue that this type of work permit is unconstitutional.^1^ Already in 2006, an Israeli court found a work permit policy that restricted migrant workers' right to change employers to be a violation of the workers' dignity and liberty and not a “least harmful measure” serving the purpose of migration control.^2^ We are not aware of such law suits in any EU Member State or against the EU for that matter. However, in the context of the recast of the EU's Single Permit Directive 2011/98, on which an agreement was reached on December 21, 2023, the right of migrants to change employers received the attention of policy makers, scholars and advocacy groups (PICUM, 2021; Politico, 2023; Weatherburn, 2023).^3^

In general, migrants' right to change employers and its limits have received little scholarly attention (but see Brock, 2020). This article contributes to filling this gap by examining the legal foundations, various modalities and implications of a limited right to change employers in the EU context. We focus on the politically contested right to change employers in the revision of the Single Permit Directive thereby illustrating the critical link between migration and labor market policies on the one hand, and migrant rights and the protection of the national workforce on the other hand. We argue that the proposals of the European Parliament would improve the rights of migrant workers considerably and work to prevent precarious employment while keeping the risks of additional labor market competition low. Yet, the Council position would have reinforced migrant precarity instead of preventing it. The final outcome of the negotiations represents a compromise the effects of which on migrant precarity are difficult to predict given the large discretion granted to the Member States. By presenting three variations of the right to change employers (unconditional, conditional, restricted) currently used in the European context, this article offers an analytical framework for future analysis and policy development and fills a gap in the literature on labor migration regulation and migrant workers' rights. This study is based on desk research of scholarly and gray literature, legal documents, and the EU legislative train on the recast of the Single Permit Directive to the extent documents have been made public at the time of writing.

The article is organized as follows. We discuss the literature on migrant precarity in view of the right to change employers and examine the respective international commitments of nation states enshrined in international human rights treaties and EU law (part 2). We then present three variants of the right to change employers as found present in EU labor migration law and selected Member States' practice. Here we focus on Germany and the Netherlands, two Member States facing labor shortages but with very different approaches to labor migration (part 3).^4^ Next, we provide insight into the state of negotiations on the recast of the Single Permit Directive and critically assess the EU's contribution to reduce migrant precarity by strengthening the right to change employers (part 4). The article ends with a number of policy recommendations (part 5) and a concluding discussion (part 6).

## 2 Immigration, precarity and the right to change employers

### 2.1 Migrant precarity and the right to change employers

In the sociological literature about transforming labor markets and welfare states, precarity and precarious employment are widely debated concepts (among many Bourdieu, 1999; Castel, 2002; Kalleberg, 2011). Precarious employment is understood as emerging from new labor market realities including the rise of part-time jobs, temporary employment and self-employment, but also from “the broader institutional structures in which work takes place” (Rittich, 2006, p. 32). Accordingly, Vosko (2010) defines precarity as “work for remuneration characterized by uncertainty, low income, and limited social benefits and statutory entitlements” (p. 2).

Precarity has also turned into a prominent topic in research on labor migration (among many Anderson, 2010; Schierup et al., 2015; Piper, 2022). Several authors have argued that precarious employment in the context of temporary migration programs is shaped by immigration regimes that “create conditions of subordination and dependence for migrant workers” (Strauss and McGrath, 2017, see also Wright et al., 2017). In the case of guest worker programmes, Ypi (2016, p. 154) notes that “workers are either typically tied to one particular work sector or the terms of their visas prevent them from changing employers”. Zou (2015, p. 141) has introduced the concept of “hyper dependence” to describe a particular tie between migrant workers and their employers “as a requirement of their legal status.” “Hyper precarity” then is caused by such ties that grant enormous control to employers thereby shaping conditions of uncertainty, temporariness and limited rights for migrant workers (Zou, 2015). One of the most radical forms of institutionalized precarity is incorporated in the Kafala system in the Arab states which binds migrant workers tightly to one employer (see for instance Salazar Parreñas and Silvey, 2021, on the underlying logics that work to discipline migrants). As legal scholars have underlined, such ties between workers and employers make migrant workers unfree and can expose them to human rights violations (Olney and Cholewinksi, 2014; Costello, 2015).

In the above contexts, migrants' right to change employers is extremely restricted or virtually absent. In employer-sponsored work arrangements, exiting one's job typically ends the right to stay and requires a new application. In other contexts, the right to change employers is conditional upon certain factors, such as the length of stay (Anderson, 2010) or the availability of professional skills (Ruhs, 2013)—thereby producing precarity for some but not, or to a lesser extent, for others.

Ruhs (2014) also points out that immigration requirements do not only make it difficult for migrants to quit jobs, but also that “the employment restrictions associated with particular types of immigration status may make migrants the more ‘suitable' workers and easier to retain in jobs that offer low wages and poor employment conditions.” Relatedly, Attas (2000, p. 90) argues in favor of a freedom to choose jobs and employers freely given that restrictions “on the freedom of occupation help to sustain artificially low wage levels […]”, with negative consequences also for natives.

However, even if migrant workers possess the right to change employers, they may be unable to comply with complex bureaucratic procedures thereby turning into overstayers (Anderson, 2010; Düvell, 2011; Costello, 2016; PICUM, 2021). Time plays an important role in this respect: Sometimes, migrant workers have to wait so long for their application to be processed that they move into *de facto* irregularity—an in-between status that puts migrants in a highly precarious situation (Farcy and Smit, 2020). We also know that the subjectively perceived (or anticipated) costs in interacting with the state can make migrants refrain from using their rights (Falkenhain and Raab, 2022). Given that the difficulties that migrants face in making use of their rights are no exceptions but commonly caused by bureaucratic and very complex systems of migration and labor market control, migrant precarity can be understood as institutionalized (Anderson, 2010).

To summarize, the elements that shape migrant precarity have been widely studied and dependence on employers has been central in that debate. We derive from the literature that the right to change employers can be strongly restricted, conditional or unconditional. Yet, in all variants, structural factors such as bureaucratic complexity can further limit migrants in their ability to change jobs.

### 2.2 International and European Union law on the right to change employers

The practice of states to restrict labor market access of admitted migrants emerged during the 20th century interwar period (De Lange, 2007). At the time, legally staying migrants had a right to access the labor market but they were only allowed to work in certain listed sectors and when no natives could be hired. As Fellman (1938) commented in 1938:

*The well-attended International Conference on the Treatment of Foreigners, held in Paris late in the year 1929, emphasized the more advanced concept of modern times, that foreigners ought to be permitted to conduct commercial transactions of every kind, to pursue all occupations except public functions […] freely and without discrimination. Such is the aspiration, at least, of a truly modern civilization*.

In fact, the right to “pursue all occupations freely” has become a human right enshrined in the International Covenant on Economic, Social and Cultural Rights (ICESCR) in 1966. According to article 6, signatory states recognize “the right to work, which includes the right of everyone to the opportunity to gain his living by work which he freely chooses or accepts, and will take appropriate steps to safeguard this right.” While the ICESCR does not make a distinction based on nationality or migration status, other treaties do so to balance the interests of migrant workers and those of receiving labor markets.

While the Migration for Employment Convention 97 of the International Labor Organization (ILO) of 1949 was silent on the right to change employer, article 14 of ILO Convention 143 of 1975 holds that the free choice of employment can only be limited for a maximum of two years or after the completion of a first fixed term contract. The application of this provision in ILO member states has led the ILO Committee of Experts on the Application of Conventions and Recommendations to call on the member states to consider the negative consequences of this restriction (Olney and Cholewinksi, 2014; Herzfeld Olsson, 2020). In the EU context this cautioning has had little impact, as we will go on to show.

The European Convention on the Legal Status of Migrant Workers, adopted by the Council of Europe in 1977, also sets time limits to tying a migrant worker to an employer. According to article 8(2), “a work permit issued for the first time may not as a rule bind the worker to the same employer or the same locality for a period longer than one year”. The treaty does not stipulate in this provision the conditions to be complied with by a worker wishing to change his job during the period of validity of the initial work permit, thus leaving that matter to the discretion of the signatory states.

The International Convention on the Protection of the Rights of All Migrant Workers and Members of Their Families (ICRMW, adopted in 1990) is less relevant for this article as none of the current EU member states has ratified it (on the EU member states' reluctance to ratify, see Desmond, 2015; on its functioning outside the EU, see Herzfeld Olsson, 2020). This Convention is rather explicit on the right of legally staying migrant workers to change employer. According to article 52(1), they “[…] shall have the right freely to choose their remunerated activity, subject to the following restrictions or conditions”. Such restrictions may include access to limited categories of employment, functions, services or activities where this is in the interest of the state and provided for by national legislation. The free choice of remunerated activity may also be restricted in accordance with its legislation concerning recognition of occupational qualifications acquired outside its territory. Specifically with regard to migrant workers whose permission to work is limited in time (such as so-called “guest workers” or seasonal workers), the receiving state may make the right to freely choose employers dependent on the passing of a certain period of time as prescribed in national legislation that should, however, not exceed two years. Yet, this time period can be extended to five years if a policy is in place granting priority to nationals—a provision that, for migrants, can result in long-term insecurity and dependence on employers.

The right of EU citizens to seek employment and to work in any Member State and for any employer has been first defined in 1968 with the Regulation 1612/68. Since 2000, it has been enshrined in the EU Charter of Fundamental Rights that EU citizens have the right to engage in work and to pursue a freely chosen or accepted occupation. This, however, does not apply to TCNs. According to art. 15(3) of the Charter, TCNs who are authorized to work in the EU enjoy equal treatment with EU citizens regarding *working conditions*. They do not enjoy equal treatment regarding the *choice of their employer* or *intra-EU mobility*.

However, as stated in Article 79 of the Treaty of the Functioning of the EU (TFEU) of 2009, the EU is obliged to “develop a common immigration policy aimed at ensuring, at all stages, the efficient management of migration flows, *fair treatment of third-country nationals residing legally in Member States*, and the prevention of, and enhanced measures to combat, illegal immigration and trafficking in human beings.” (italics added).

Finally, albeit soft law, the UN Global Compact on Safe, Orderly and Regular Migration, that was signed in December 2018 by 19 EU Member States considers administrative complexity as a barrier to changing employers. According to recommendation nr. 6, all signatories need to “[d]evelop and strengthen labor migration and fair and ethical recruitment processes that allow migrants to change employers and modify the conditions or length of their stay with minimal administrative burden […]”.

In sum, this brief review – with no pretense of completeness but sufficient for our purpose – has shown that the right of migrants to change employer and Member States' commitment for fair treatment of migrant workers can be derived from international treaties. However, much discretion is given to nation states opening up the possibility of implementing the right to change employers differently and limiting it. Although the right of nation states to limit migrants' rights and reserve certain rights to natives has been considered as an aspect of sovereignty (Dauvergne and Marsden, 2014), recent human rights scholarship contests the exclusion of migrants from certain rights by questioning the historical interpretation of international law in legal scholarship (Spijkerboer, 2021). It seems that restrictions of the right to free choice of employment have found their way into international human rights law thereby exacerbating fundamental inequality between citizens and non-citizens that it set out to mediate.

## 3 Three variants of the right to change employer

Taking the above considerations as a starting point, we distinguish between three variants of the right to change employers currently in place in the EU and its Member States. As Table 1 illustrates, the right of migrant workers to change employers can be unconditional, conditional or restricted.^5^ A key concept in, and one justifying the use of, the different variants is the so-called “priority workforce” in the Member State of first admission, i.e. the group generally consisting of nationals, EU nationals and long-term residing TCNs that employers must give preference over new hires when filling posts (Robin-Olivier, 2016). The key tool to decide whether a job can(not) be filled with priority workforce is a “labor market test” that typically consists of a “priority check” together with an “equivalence check” of the labor conditions to ensure that foreigners are not employed under less favorable conditions than nationals in similar positions. The labor market test might be supplemented by other (employer-related) requirements that fall outside the scope of our analysis (see European Migration Network, 2021, providing a mapping of labor market tests in 22 EU member states). Table 1 summarizes the three variants, their modalities and implications for migrant workers.

**Table 1:** Variants, conditions, and consequences of the right to change employers.

**Right to change employer**	**Conditions and modalities**	**Consequences and potential risks for migrants**
Unconditional	• Workers belonging to priority workforce, labor market access like nationals and EU citizens	• In-country labor market mobility
Conditional	• Conditions can regard availability of priority workforce, waiting time, sector or profession of first employment, country of origin, region of first employment, or limited number of changes •Administrative procedures can vary from notifying the authorities to asking for and awaiting formal approval •Procedure to be initiated in-country by the migrant worker or the new employer and/or reporting obligation for the former and/or new employer	• Fulfilling certain conditions (e.g., waiting time) can lead to status improvement (toward unconditional right) •Risk of gaps in legal residence in case of complex/lengthy administrative procedures or negative labor market test
Restricted	• New admission procedure required: testing all conditions as for first admission, including labor market test for available priority workforce •Possibly barred from in-country application	• Risk of gaps in legal residence in case of lengthy procedures or negative labor market test •Migrants forced into (temporary) irregularity or to leave the country •Migrants discouraged from changing jobs

An **unconditional right** to change employers applies to legally resident TCNs admitted for a variety of reasons. Their migration status entitles them to equal treatment with nationals and EU citizens, *and* qualifies them as priority workforce. As a consequence, they can change jobs in the host country without undergoing a labor market test, and in case of competing applications, would be given preference over TCNs that are part of the non-priority workforce. In the EU context, this group includes holders of a refugee status under the EU Qualification Directive 2011/95 and those with permanent status under the EU Long-Term Residence Directive 2003/109 or national law. Recently graduated international students get a minimum of nine months unconditional access to the labor market and thus a right to change employers by way of a search period to find a job in the EU (article 25 Students & Researchers Directive 2016/801). Moreover, again depending on national law, some family migrants, e.g., spouses of nationals under the Family Reunification Directive 2003/86 or spouses of Blue Card holders benefit from an unconditional right to access the labor market and thus an unconditional right to change employer. For TCNs coming to the EU for work, an unconditional right to change employer is rare, yet it is not unseen. For instance, the Spanish model as described by Weatherburn (2023) grants full labor market access to single permit holders upon arrival. However, to be eligible for an extension of the permit, the migrant worker needs to prove legal employment for at least three months, making the scheme conditional in a different way.

A **conditional right** to change employers is more commonly applicable to TCNs who come to the EU for work purposes and do not (yet) qualify as priority workforce. The most far reaching condition for a change of employer is a new labor market test. However, depending on their migration entry category, status and skill level, TCNs with a conditional right to change, might enjoy exemptions from (parts of) the labor market test. Blue card holders, highly qualified and well-paid migrant workers, are a case in point. Under the revised EU Blue Card Directive 2021/1883, Member States are allowed to require that, during the first twelve months of legal employment as an EU Blue Card holder, any change of employer needs to *be communicated* to the competent authorities and that the competent authorities may carry out a *check of the labor market situation*. Yet, after that twelve-month period, the labor market test needs to be waived, and Member States are only allowed to require a notification of a change of employer (Recital 42 Blue Card Directive, Article 15 Blue Card Directive 2021/1883/EU). The rationale behind such a waiver of the restrictions is that high-skilled migrant workers have paid into the system and supposedly “deserve” more flexibility after a certain time period. Still, the new job needs to fulfill certain conditions, such as meeting a salary threshold or corresponding to higher professional qualifications.

TCNs may also benefit from the right to change employers if they fulfill certain conditions as defined by national law. These conditions may relate to their skills, (shortage) occupations or sector of employment. Even if they have a right to change jobs as such, this can still be limited, for instance only allowing job changes within the sector or profession of first employment. There can be regional restrictions, too, according to which job changes are possible, yet a stay within the region of original employment is required.

Finally, as Anderson (2010, p. 306) has rightly noted, the “length of stay has implications for rights-based claims.” This is also true here: The time of legal employment in the Member State of first admission can be a relevant condition, meaning that migrants acquire an unconditional right to change after a certain time period. According to international law (see Section 2.2.), this is commonly after one or two years but should definitely be the case after five years. In Germany, labor migrants from third countries are allowed to change jobs without restrictions after two years of legal employment in the country (Article 9, German Employment Ordinance), while this is only possibly after five years in the Netherlands (Article 4, Dutch Foreign Nationals Employment Act).

Similar to the conditional variant, a **restricted right** to change employers applies to legally resident TCNs that come to the EU for work purposes and do not belong to the priority workforce in the host country. In this variant, a change of employer is possible but it requires going through the same procedure as for first admission again. If in-country applications are not permitted, this could even mean that the TCN has to return to the country of origin and apply for a new work permit – which *de facto* is no longer a change of employer. As we will show later, this was a contested topic in the negotiations on the recast of the Single Permit Directive (see Section 4.2).

At EU level, a restricted right to change employers is for instance granted to seasonal workers. According to the Seasonal Workers Directive 2014/36/EU [article 15(3)], the EU Member States shall allow for a one time change of employer within the maximum duration of stay (which is five to nine months according to article 14), provided that the admission criteria continue to be met. Member States *may* refuse the application for a change in case there is priority workforce available [article 15(6)], potentially nullifying the right to change. Unlike the Blue Card where a notification of a job change suffices, the new employer of the seasonal worker has to lodge a new permit application with the competent authorities. According to recital 31 of the Seasonal Workers Directives, the opportunity to change jobs should “serve to reduce the risk of abuse that seasonal workers may face if tied to a single employer and at the same time provide for a flexible response to employers' actual workforce needs.” Yet, as described, migrants' right to change is restricted (Rijken, 2015; Zoeteweij, 2018; Bregiannis, 2021).

In Germany, migrants employed on the basis of the reformed Western Balkans regulation [Article 26(2) German Employment Ordinance], that is open to workers irrespective of their formal skill level, enjoy only a restricted right to change employers. The waiver of the labor market test after two years (see under the conditional variant, article 9 German Employment Ordinance), does not apply here, indicating a clear legal disadvantage for potentially lower-waged workers. In the Netherlands, changing employers is always an option, although most often the restricted variant applies, even for highly skilled migrants. This means that the new job (or employer) must qualify for a right to stay, and the whole application procedure is to be followed again, including labor market testing if applicable. This is highly uncertain and time consuming, with procedures lasting up to nine months, if successful at all. Only if for instance a highly skilled *knowledge migrant* changes to an employer already recognized as a sponsor, this procedure can be finalized in a couple of weeks. Otherwise, it is highly unlikely that the procedure is finalized within the period of three months granted for a change of employers. This means that migrants have to prepare job changes well in advance, which likely disincentives them. It also shows that a restricted right to change employers can have different implications for migrants depending on their status or skill levels, and the respective administrative procedures in place, with unequal treatment on these grounds as a result.

Another recent example concerns migrant nurses who have been recruited for the Dutch labor market and complained of feeling abused. They had not been properly informed of the fact that within this project, which was approved by the Dutch government, they had no chance to choose their first employer or change employer later on in the four year project.^6^ Furthermore, in Germany and the Netherlands, even short income gaps resulting from job changes (e.g., when, in case of involuntary unemployment, a new employer is not immediately found, or when that employer fails to issue the working contract soon enough) can potentially be held against migrant workers after five years of legal residence, when they apply for long-term residence. In the case of a Brazilian worker in the Netherlands, such an income gap elongated the insecurity of residence for another five years—a consequence that the Dutch Court he had turned to did not consider disproportionate.^7^

To summarize, the right for TCNs to change jobs varies and is highly fragmented at the EU and national levels (see also Weatherburn, 2023). The fragmentation across Member States shows how discretion to administer that right has been used (see Section 2.2) with little attention for equal or fair treatment of the migrant workers involved. Two findings are worth noticing: firstly, as we have seen, the right of TCNs to change employers is typically conditioned or restricted. The limitations mirror a broader trend: The right to change jobs is often a selective right, offering more and quicker flexibility to the (highly) skilled workers considered as “deserving” and less protecting low-waged migrants admitted when in demand but just as easily discarded when the labor market changes. In the current labor market situation in the EU and its Member States, with a high level of labor shortages, a renewed labor market test upon job change might seem unproblematic at first view. However, were the labor market to become less overheated, migrant workers admitted temporarily for the purpose of work would likely be the first to lose their job and find themselves unemployed, (eventually) forced to leave the EU, or find themselves in an irregular position if their (new) employer cannot prove that there is no priority workforce. Moreover, even in a positive labor market situation, a conditional or restricted right to change employers makes the chances of a job change unpredictable for the migrants themselves. Complex and time-intensive procedures pose a risk of gaps in legal residence, or periods of irregular stay in-between jobs (see Table 1). The uncertainty might discourage migrant workers from considering a job change at all, for instance for career purposes but also to quit low quality jobs or abusive work relations (Falkenhain and Raab, 2022).

Secondly, the practice review has shown that intra-EU mobility rights—changing employers across EU border—are hardly given (see also Pascouau, 2013). TCNs with an unconditional right to access the labor market in one EU member state may regress to a conditional or heavily restricted right in another member state. While the revised Blue Card Directive enhances intra-EU mobility (De Lange and Vankova, 2022), other TCNs moving for work purposes from one into another EU Member State will be confronted with high hurdles for admission and job changes, or likely move into irregularity (Della Torre and De Lange, 2018). Such hindered mobility might decrease the attractiveness of the EU in the eyes of prospective migrants [COM(2022)656]. In times of acute labor shortages, enhancing the right to change employer in one Member State as well as across the EU would seem a viable labor market policy option, allowing for a better allocation of workers present in the EU.

But even without labor shortage, the relevance of removing the obstacles to change employers has been recognized in international and European law (see Section 2.2). Still, as the revision of the EU Single Permit has shown, it is a right that has to be fought for in the political arena. The negotiations are an ideal opportunity to examine the contested right to change employers and ask: What conditions will be attached to the right to change employers? What procedures are foreseen to enforce it? Will the reform reduce migrant precarity and improve equal treatment compared to current practice, or will it limit migrant mobility for the supposed good of national labor markets and the national workforce?

## 4 The right to change employers in the EU Single Permit Directive

### 4.1 Background

The European Single Permit Directive (SPD) 2011/98 was adopted in 2011. According to the Commission, the Directive has been fully transposed into national law by 25 Member States. Denmark and Ireland are not subject to it. Due to a lack of data from several Member States, the reported number of permits do not give a full picture.^8^ Between 2016 and 2022, the highest share of single permits was granted for employment purposes followed by family reasons. Over 90 percent of all recorded permits in that period had a validity of twelve months or more (Eurostat, 2022).

The objective of the Single Permit Directive is twofold. Firstly, it aims to facilitate the procedures for third-country nationals to obtain a single permit for the purpose of work and stay in the European Union. As an umbrella directive, it is silent on the conditions of entry, covered in other Directives (e.g., Seasonal Workers' Directive 2014/36/EU, Blue Card Directive 2021/1883/EU), or national laws of the EU Member States. Secondly, it lays down a common set of rights to ensure equal treatment of TCNs with nationals in terms of working conditions, including pay and dismissal as well as health and safety at the workplace, freedom of association, training and education, recognition of diplomas according to national law, and social security and tax benefits (article 12 SPD). In 2019 and 2020, the European Commission issued evaluations of the Directive according to which the equal treatment provisions are interpreted and implemented differently by Member States, and the protection of migrant workers against labor exploitation is insufficient (Ahamad Madatali, 2022). The Commission announced to address these and other problems in a revision of the Single Permit Directive as part of the 2020 New Pact on Migration and Asylum (see De Bruycker, 2020, for an evaluation of the pact).

The 2011 Single Permit Directive is silent on the right to change employer, leaving this to the discretion of the EU Member States. The Impact Assessment found that Member States' practices largely differ in terms of options to change employers [COM(2022)656: 18-19]. For instance, the procedures for renewing a permit vary greatly and take from 30 to 120 days [COM(2022)656: 82], thus leading to extended periods of uncertainty for applicants. Some stakeholders (e.g. the European Economic and Social Committee, the European Migration Forum, the NGO PICUM) emphasized that migrant workers' dependence on their employer and their inability to change jobs when waiting for the renewal of their permit run counter to the fair treatment objective and raise the risk of exploitation. Similarly, Weatherburn (2023) has examined the barriers experienced by single permit holders to change their employer in Belgium, the Czech republic and Spain. She also stresses the risk of vulnerability stemming from a strong dependency on the first employer. The same worry is at the heart of the critique voiced by the NGO Politico (2023) according to which single permits lock migrants into jobs, regardless of occupation.

### 4.2 The revision of the Single Permit Directive

The legislative reform process of the Single Permit Directive started with the European Commissions' recast proposal presented in April 2022 [COM(2022)655] (Orav, 2023). The recast proposal provided, amongst others, that TCN workers should be entitled to change employers *during the validity of a single permit* (Article 11, paragraph 2). If, for instance, a worker from Vietnam receives a one-year single permit in Germany and after eight months wants to change employer, this is within the validity and should thus be allowed. However, article 11(3) of the recast proposal sets the parameters for EU Member States to restrict the right to change. Accordingly, within the period of validity of a single permit, Member States may (a) require that a change of employer should be communicated to the competent authorities in the Member State concerned, in accordance with procedures laid down in national law, and (b) require that a change of employer is made subject to a check of the labor market situation. Furthermore, the right of the single permit holder to pursue a change of employer may be suspended for a maximum of 30 days while the Member State concerned checks the labor market situation and verifies that the requirements laid down by Union or national law are fulfilled. Thus, depending on national law, this is a conditional or restricted right to change employers. In fact, it allows for a repetition of an already performed full labor market test with priority workforce check and/or an equivalence check. The discretion granted to the Member States in this proposal offered little hope for a harmonized approach across the EU member states. The European Commission also proposed that whichever way the Member States decide to implement their discretion, they have to decide within 30 days either allowing the switch, or not. This 30-day period would be considerably shorter than the standard period of four months EU Member States have for taking a first entry decision (Article 5(2) original and recast proposal). Importantly, the Commission also proposed that the Member States shall allow for in-country applications when migrant workers who are legally employed in the EU seek to amend their permit, for instance to change the employer [Article 4(1) recast proposal]. In case of a negative result of a priority workforce check or an equivalence check, the worker would be forced to remain with the old employer and find another job that will be approved. If not successful in finding a new job before the expiration of the validity of the single permit, the worker would need to leave the country. While this proposed text would have slightly improved TCN workers' right to change employers compared to current practice in some Member States, the Commission's recast proposal still catered to the Member States' wishes to protect the interests of the national workforce by allowing a labor market test. In addition, it would have single permit holders less mobile on the labor market than the highly qualified Blue Card Holders as discussed above (see Section 3). Instead of closing the gap between migrants of different skill and wage levels, it would perpetuate the unequal treatment of the two groups.

The negotiations within the European Parliament (EP) were concluded on 14 April 2023.^9^ Like the Commission proposal, the EP amendments make explicit reference to the *right* to change employers. Some MEPs suggested incorporating an unconditional right to change employer in Article 11(1)(d), (Amendment 72), but this was not included in the final EP compromise.^10^ The EP amendments illustrate the two labor market policy goals at stake. Firstly, while the Commission's proposal gave discretion to the EU Member States regarding the reporting of a change, the EP proposal *obliges* the Member States to require that (1) a job change is communicated by the new employer prior to the commencement of the new employment (Proposed recital 22a) and (2) the new employer provides information on the type of work, working hours and remuneration [Article 11(3) Amendment 76]. This communication (unlike the notification under the Blue Card Directive mentioned above) is not the same as a full permit application but shall allow the Member States to conduct an equivalence check to ensure decent working conditions and protect the migrant workers from potentially exploitative employment. Secondly, while the Commission proposes to allow a full priority workforce check, the EP deleted this option (Amendment 78). However, it does foresee a labor market test when the change of employer involves a change of sector *and* only when the Member State generally carries out labor market tests for single permits (Amendment 80). This conditional variant of the right to change employers aims to protect the interests of the national labor market and priority workforce *but only* if the migrant switches sectors, meaning a previously performed labor market test may not be repeated if the TCN remains in the original sector. Compared to a general labor market test as proposed by the Commission, the European Parliament's proposal would reduce legal uncertainty for migrant workers, at least for those not changing the sector of first employment, and is more considerate of the administrative burden experienced by the new employer than the Commission's proposal. Thirdly, both the Commission and the EP want the labor market testing to happen quickly: the national authorities will have 30 days to conduct the tests. Novel is the EP amendment that would have the permission to change employers *granted* if no decision to refuse the change is handed down in time. This would avoid long-term uncertainty for migrant workers due to capacity constraints in national administrative systems.

The Council representing the Member State governments entered the negotiations in June 2023 (COM 10363/23) with a strongly restrictive position regarding the right to change employers. The Council position ties TCN workers to their employers more than the Commission's proposal envisaged, and does not oblige the Member States to allow for in-country applications to amend a permit. This could encourage some EU member states to ask migrants to leave the country before being able to apply for another job. In recital 34, the Council is still largely positive about changing employers, but the specific proposals go into another direction. It agrees that TCN workers should be allowed to change employer during the validity of the single permit. The applicable procedure could be either a notification of the change, possibly with certain conditions for a change of employer in place, or a full application procedure and a check of the labor market situation. The choice for either model – the conditional or the restricted variant – is left to the Member States, potentially leaving many single permit holders across the EU tied to their employer and facing severe restrictions to change jobs.

In addition, the Council proposes that the Member States can (but would not be obliged to) set conditions with respect to changing occupation, the occupational sector or the “substantial characteristics of the employment”. The Council moreover wants Member States to be able to set a time restriction during which job changes are prohibited, both to avoid that workers are lured away by abusive employers making false promises, and to protect the interests of the first employer who has invested resources in the recruitment and training of TCN workers. According to the Council proposals, the time restriction can be waived “in exceptional and duly justified cases, for example in case of exploitation of the single permit holder or if the employer fails to meet its legal obligations in relation to the single permit holder.” This shows awareness of the risk of abuse and the importance of freedom of choice. However, practically, we wonder how useful this exception is, as before a legal case of exploitation is proven to be one that “duly justifies” waiving the time restriction, in all likelihood, a lot of time has passed. Finally, and this would nullify the right to change if agreed upon, the Council wants to enable the Member States to withdraw a single permit after a period of (at least) two months in the event of unemployment [Article 11(3) Council position]. Such a tight timeframe of two months together with the requirement of a full labor market test would likely discourage migrant workers from leaving abusive jobs and entering a phase of unemployment in order not to jeopardize their right to stay, as also described by Weatherburn (2023). At the same time, the Council deletes the fast-track procedure of 30 days to (dis)approve job changes foreseen by the EP and Commission, and instead proposes a period of 90 days [Article 11(2) sub a Council position]. With this, the Council potentially prolongs phases of irregular stay of those who are in-between jobs. As we know, lengthy administrative procedures can have various adverse effects: It might make job changes unattractive for migrant workers who fear to lose their right of residence. Also, it does not offer flexibility to employers to recruit needed workers, for instance in times of acute labor shortages.

In sum, if the Council's position became law, this would reinforce the precarity of TCN workers considerably by creating uncertainty for migrant workers and reducing their freedom to exit employment while remaining in the country. The many references to national law in the Council's position illustrate a lack of political will to harmonize the field. While at the time of writing, the trilogue negotiations between the Commission, the EP and the Council were ongoing, members of the European Parliament told us that the Spanish rapporteur Javier Moreno Sánchez would like to see the recast adopted by the end of 2023 during the Spanish presidency of the Council. In this they succeeded. An agreement was reached on December 21, 2023, just before finalizing this text.^11^

The compromise text explicitly mentions a right to change employer in the new article 11(2). Member States are obliged to allow a single permit holder to change employer, yet they may set conditions according to article 11(3). Member States may for instance carry out labor market tests when they generally do so (the restrictive variant), and they can require the workers to stay with the first employer for a minimum period. The foreseen six months period is clearly shorter than the one year period suggested by the Council, but the compromise is much stricter compared to the Commission's and the Parliament's position that did not foresee such a possibility to bind workers to their first employer. According to the compromise text, the migrant worker must be allowed to change before the expiration of a minimum period in ‘duly justified cases of a serious breach by the employer of the contractual terms and conditions'. As discussed above, this exception is well-intentioned, but it remains unclear whether it can really benefit migrant workers in case of time-intensive administrative procedures and court decisions. The compromise text further states that the new employer has to notify the competent authorities in accordance with national law. After such a notification, the Member States have 45 days to verify if the conditions for changing are met (article 11(3, last paragraph) and to (dis)approve the change. This period may be extended for another 15 days 'in exceptional and duly justified circumstances' (article 8(3) sub b), a provision that is very vague. In this respect, the recast of the Single Permit Directive is a step forward compared to the status quo but does not reach the ambitions of the Parliament and the Commission to speed up the decision-making. Finally, two provisions are a positive sign: Recital 22a rightly clarifies that the Member States may not consider it a 'change of employer' when the conditions of employment change, for instance, the habitual place of work, or the remuneration. And according to article 1a, in-country applications for a change of employer are foreseen. To summarize, although the objective of the recast of the Single Permit Directive was to further harmonize EU migration law, the provisions regarding the right to change employers grant much discretion to Member States. It thus remains to be seen which variants of the right to change will be implemented at the national level, and where migrant precarity will be substantially reduced.

## 5 Policy recommendations: reducing precarity, not reinforcing it

The different positions on the revision of the Single Permit Directive have revealed the potential for strengthening migrants' right to change employers but also for clear setbacks. In line with Zhang et al. (2022), we call on policymakers to humanize labor migration and reassess the impact of legal decisions on work relations and migrant lives. We suggest this can be done in the following five ways.

Firstly, we consider the restricted right to change employers disproportionate as it unduly binds migrant workers to their first employer and disincentives job changes. The European Commission and more so the European Parliament have proposed some important legislation to offer more protection and improve migrants' right to change employers compared to current practice. Yet, the Councils' proposal remained far behind. The compromise which allows for six months of a restricted or near absent right to change does not enforce the fair treatment of migrant workers in line with international human rights standards and commitments. As the compromise text shows, the EU has not shied away from laying the basis for the restrictive variant. It is now up to the Member States to avoid this variant when implementing the recast. We see the advantage of — temporary—sectoral restrictions as foreseen in the European Parliament's amendments. They aim to protect the national workforce from (feared) competition while not tying migrant workers to a specific employer. The right to leave low-quality or indecent employment while not jeopardizing the right to stay might justify this solution even if migrant mobility is still limited (Ruhs and Chang, 2004; Brock, 2020). Refraining from further restrictions and from granting too much discretion to Member States in that regard could also work to avoid possible law suits as the one successfully engaged in Israël and pending in Canada (see Section 1).

Secondly, the recast is a missed opportunity to strengthen intra-EU mobility for single permit holders. Situations of labor market crises that encompass national borders, such as the COVID-19 pandemic, call for (more) dynamic concepts and ways to facilitate job changes to respond to quickly arising labor shortages in certain sectors. More generally, intra-EU mobility could also help respond to long-term demand for labor due to demographic changes in many EU Member States. We thus recommend the EU to improve the intra-EU mobility rights for TCN workers. This recommendation could also target the recast of the Long-Term Residence Directive which is currently under negotiation as well.^12^ The Council wants to grant the Member States the discretion to perform labor market tests in case of intra-EU mobility, which we would advise against from a labor market and migrant rights perspective.

Thirdly, regarding the application of the conditional and restrictive variants, the modalities of the labor market test are crucial. As we know, labor market tests are not applied uniformly within the EU (European Migration Network, 2021). Nor have the benefits of priority workforce checks been proven scientifically. The instrument is complex in its administration and its efficiency is uncertain as it is often difficult for the responsible agencies to prove that there are preferential employees and that the position can actually be filled (Adunts et al., 2023). Against this background, the European Commission should draft guidelines – like those regarding the interpretation of the Family Reunification Directive – to explain what could (and what should not) be included in a labor market test, and specifically in a priority workforce check considering the obligation to ensure the fair treatment of migrants as enshrined in international human rights treaties. We argue that the EP's proposal, that has not been integrated into the final compromise text, could pave the way for a more tailored labor market test. A wider use of the equivalence check (of labor conditions) decoupled from a priority workforce check could lessen the administrative burden, speed up processes, and work to protect migrant workers instead of penalizing them.

Fourthly and relatedly, besides the *right* to change employers, the *procedure* that applies is key to it being a serious option at all. Here, we highlight the importance of timing in the process of job changes, as speeding up administrative procedures can decrease institutionalized uncertainty (Anderson, 2010). The Commission and the Parliament proposed 30 days where the Council proposed 90 days, which would have been a clear backward development if taken on board. They agreed on 45 days with a possible extension to 60 days in exceptional circumstances. Even the midway compromise of 45 to 60 days for the decision to be taken is long if, in the meantime, the migrant is to remain without a source of income in case the previous job has already been terminated. Here, the risk of labor migration law causing irregularity (i.e., staying without a residence permit) is high. This risk should alert the EU Member States to keep the process short. In addition, the EP's proposal to consider the permission granted if no decision is taken in time rightly places responsibility on the Member States instead of penalizing migrant workers. Interestingly, a respective provision has not been incorporated in the final agreement (article 5(2) Recast Single Permit Directive) leaving room for more divergencies at the national level.

Beyond a purely black letter law perspective, we know that inequality between migrant workers is also produced due to implementation. Opaque administrative procedures and lack of knowledge about rights can discourage migrant workers from claiming their rights. That said, even when the right to change employers exists and conditions improve, and if processes would speed up according to the Commission's or the Parliament's proposals, uncertainty may persist and keep (some) migrant workers immobilized. This insight draws attention to the dire need to increase transparency, simplify administrative procedures and ensure language-sensitive communication with national authorities. These are and remain important tasks for EU Member States.

Fifthly and finally, employers also play an important role given their financial, social and timely investments in getting migrants to Europe, in providing onboarding and other integration measures. Employers might expect returns for their early investments. From this perspective, more rights for migrants could, in the worst case, encourage employers to impose informal or formal contractual hurdles to job changes through repayment or commitment clauses on their workers. In our view, this risk should alert Member States to better monitor employers and check labor conditions over time and not only prior to employment, demand fair recruitment practices, and provide accessible (free) legal aid to migrant workers. Employers should step up their responsibility in human rights enforcement, too, to set positive incentives for migrant workers to stay.

## 6 Conclusion

Facing labor shortages, national governments commonly aim to attract foreign workers while at the same time trying to prevent labor market competition for the national workforce. At the heart of the intersection of these migration and labor market policy goals is the question of whether and under what conditions migrant workers from third countries, once admitted, may change their employer to quit exploitative employment or for career reasons. Typically, migrants' right to change employers is limited, with various consequences for work relations and migrant lives. The aim of this article was to analyze the legal foundations, modalities and implications of a limited right to change employers in the EU context. Building on the literature on employer dependency and migrant precarity, as well as on a review of the right to change employer and its limitations in international and EU law (Section 2), this article has presented three policy variations of the right to change employers currently in place in the European context (Section 3). In so doing we have contributed to filling a gap in the literature on labor migration, labor market regulation, and migrant workers' rights.

The article has then focused on the contested right to change employers in the revision of the EU Single Permit Directive (see Section 4). Our analysis of the position of the Council leads us to conclude that EU Member States do not intent to live up to international human rights standards of fair and ethical labor migration and of equal treatment with respect to the right to choose one's employment. With its proposals, the Council would have failed to improve migrants' right to change employers. Although the EP proposals are most far-reaching, they still resonate the need to protect the national workforce. The consensus reached on December 21, 2023 may reduce precarity but might also generate irregular stays, i.e. stays without legal residence between jobs (see also Weatherburn, 2023). This is the opposite of the objective of Article 79 of the TFEU, on which the Single Permit Directive builds, to offer fair treatment to third country nationals in the EU. With our policy recommendations (see Section 5), we call on the EU and its Member States to humanize its labor migration policy and to prevent undue restrictions to the freedom to exit employment.

The EU debate has so far not addressed or tried to dismantle the prevalent dichotomy between the so-called high-skilled and lower-skilled or low-waged migrants. While the former are commonly seen as *deserving* rights-based mobility (among many, Chauvin et al., 2013), deservingness is rarely highlighted for the latter. Interestingly, the dichotomy is not as pronounced in the Netherlands where highly skilled migrants can be just as cut-off from the opportunity to change employer as low-waged workers. Indeed, the brief comparison between Germany and the Netherlands has highlighted a difference in the approach toward skilled workers and those with practical skills but often little formal qualifications. The new Skilled Immigration Act adopted in Germany in July 2023 increases the gap between the two countries in further facilitating job changes and intra-EU mobility for skilled and highly skilled workers (Deutscher Bundestag, 2023).^13^ While the recast of the EU Single Permit Directive could have been an opportunity to harmonize the EU Member States' positions toward migrant workers of different skill-levels after admission, the Council position suggested that the Member States were not too interested in such a harmonization.

Finally, we hope our contribution ignites future research into the multiple and sometimes conflicting policy goals identified and the design and functioning of migrant workers' rights beyond entry conditions. Our analytical framework that distinguishes between three different variants of the right to change employers has proven valuable to explore hurdles and facilitators of job changes as well as potential risks for migrant workers, and could be used for comparative purposes in future studies - inter alia in the scientific evaluation of the recast of the Single Permit Directive. The right to change employers has only recently started to receive substantive attention in EU policy debates. The latter win strength with solid empirical evidence on the experiences of all actors involved—migrant workers, employers, and administrative actors—with formal migration and labor rights, administrative procedures, and practicalities.

## Author contributions

TDL: Conceptualization, Investigation, Writing – original draft, Writing – review & editing. MF: Conceptualization, Investigation, Writing – original draft, Writing – review & editing.

## Footnotes

^1^https://www.cbc.ca/news/canada/montreal/montreal-based-workers-rights-group-files-class-action-against-closed-work-permits-1.6969241, last visited 27 November 2023.

^2^See the case *Kav LaOved Worker's Hotline v. Government of Israel*.

^3^See also the Joint Statement of 18 European networks and organizations on the revision of the Single Permit Directive, https://www.etuc.org/sites/default/files/document/file/2023-11/Joint%20Statement_Revision%20of%20the%20SPD_15%20Nov.pdf, last accessed: 5 December 2023.

^4^Germany has a relatively open labor migration policy and develops it further while the Netherlands follows a restrictive policy towards non-EU labor migrants (Kolb, 2017; Brücker et al., 2020; De Lange, 2023).

^5^Excluded from the visualization are intra-corporate transfers under Directive 2014/66/EU that constitute a very specific tie between workers and employers, and the EEC-Turkey Association Agreement of 1960 which sets specific (less stringent) conditions on the right to change employer for Turkish employees.

^6^Indonesian nurses recruited for the Dutch labor market complained about the lack of freedom to change their employer, restricted by recruiting agencies: “Indonesische verpleegkundigen worden hier uitgeknepen: ‘Ik voel me opgelicht'.” (Indonesian nurses are abused here: 'I feel betrayed'), https://www.nu.nl/binnenland/6272816/indonesische-verpleegkundigen-worden-hier-uitgeknepen-ik-voel-me-opgelicht.html, 20 July 2023.

^7^District Court The Hague, 16 August 2022, ECLI:NL:RBDHA:2022:14531.

^8^The reported number of permits between 2016 and 2021 is far below real numbers. However, it indicates some general trends including a peak (of 3 million new and renewed single permits) in 2019, followed by a slight drop likely due to the COVID-19 pandemic in 2020.

^9^EP Compromise Amendments COM(2022)655 – A9-0140/2023 delivered by rapporteur Moreno Sánchez of the LIBE Committee. Jongerius was rapporteur of the associated Committee on Employment and Social Affairs.

^10^Another critical point for debate is the scope of the Directive and whether it will cover the rights of displaced people from Ukraine employed in the EU. On that topic, see Committee Meijers, https://www.commissie-meijers.nl/wp-content/uploads/2023/07/230714-Meijers-Committee-Comment-on-End-of-Ukraine-Temporary-Protection-Scheme-.pdf.

^11^Council Document 17041/23, 21 December 2023, Outcome of Proceedings, Inter institutional File: 2022/0131(COD).

^12^Proposal for a DIRECTIVE OF THE EUROPEAN PARLIAMENT AND OF THE COUNCIL concerning the status of third-country nationals who are long-term residents (recast), COM/2022/650 final. On the Councils' position of 28 November 2023 see https://data.consilium.europa.eu/doc/document/ST-16000-2023-INIT/en/pdf, last accessed 7 December 2023.

^13^See also: https://www.migrationsrecht.net/nachrichten-auslaenderrecht-politik-gesetzgebung/fachkraefteeinwanderungsgesetz-regelt-beschaeftigung-umfassend-neu.html (last accessed: 12 December 2023)
